# Recent Advances in Hepatitis C Virus Cell Entry

**DOI:** 10.3390/v2030692

**Published:** 2010-03-08

**Authors:** Birke Bartosch, Jean Dubuisson

**Affiliations:** 1 INSERM, U871, 69003 Lyon, France; 2 Université Lyon 1, IFR62 Lyon-Est, 69008 Lyon, France; 3 Hospices Civils de Lyon, Hôtel Dieu, Service d’hépatologie et de gastroentérologie, 69002 Lyon, France; 4 Université Lille Nord de France, F-59000 Lille, France; E-Mail: Jean.Dubuisson@ibl.fr (J.D.); 5 CNRS, Institut de Biologie de Lille (UMR8161), F-59021 Lille, France; 6 Institut Pasteur de Lille, F-59019 Lille, France

**Keywords:** hepatitis C virus, cell entry

## Abstract

More than 170 million patients worldwide are chronically infected with hepatitis C virus (HCV). Prevalence rates range from 0.5% in Northern European countries to 28% in some areas of Egypt. HCV is hepatotropic, and in many countries chronic hepatitis C is a leading cause of liver disease including fibrosis, cirrhosis and hepatocellular carcinoma. HCV persists in 50–85% of infected patients, and once chronic infection is established, spontaneous clearance is rare. HCV is a member of the *Flaviviridae* family, in which it forms its own genus. Many lines of evidence suggest that the HCV life cycle displays many differences to that of other *Flaviviridae* family members. Some of these differences may be due to the close interaction of HCV with its host’s lipid and particular triglyceride metabolism in the liver, which may explain why the virus can be found in association with lipoproteins in serum of infected patients. This review focuses on the molecular events underlying the HCV cell entry process and the respective roles of cellular co-factors that have been implied in these events. These include, among others, the lipoprotein receptors low density lipoprotein receptor and scavenger receptor BI, the tight junction factors occludin and claudin-1 as well as the tetraspanin CD81. We discuss the roles of these cellular factors in HCV cell entry and how association of HCV with lipoproteins may modulate the cell entry process.

## 1. Introduction

Chronic hepatitis C is a leading cause of chronic hepatitis, cirrhosis, and liver cancer. Worldwide, there are currently more than 170 million individuals infected with the hepatitis C virus (HCV), with the majority of cases remaining undiagnosed and untreated [1]. The available therapy, a combination of pegylated interferon and ribavirin, has limited efficacy and significant side effects; drugs that specifically target viral enzymes are yet to reach the market.

**Figure 1:**
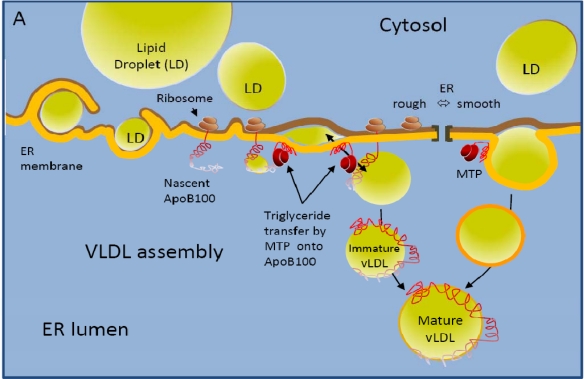

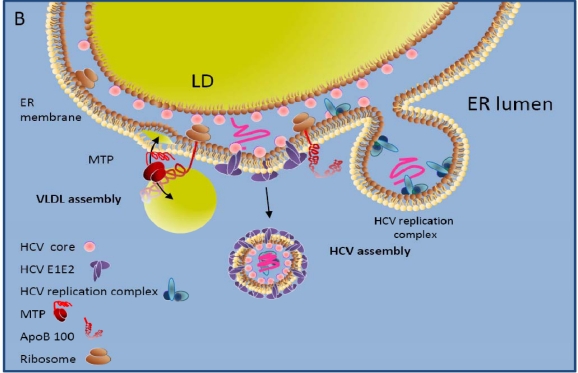
**VLDL and HCV assembly. A. VLDL assembly.** Upon translation, nascent apolipoprotein 100 (apoB100) translocates through the endoplasmatic reticulum (ER) membrane. The protein is very unstable and subject to proteasomal degradation, unless it is rapidly lipidated. Lipidation of nascent apoB can occur by two different mechanisms. Either free MTP binds nascent apoB and subsequently extracts lipids from the membrane and transfers them into a hydrophobic pocket of the nascent apoB polypeptide. MTP molecules that are not physically associated with apoB can further assist this step and the pocket may serve as a nucleation site for lipid deposition. Several rounds of this process will result in extensive lipidation of apoB until the apolipoprotein/lipid complex is large enough to bud off from the rough ER membrane in the form of an immature VLDL. Alternatively, MTP associated with lipid vesicles or droplets may bind to apoB and provide a lipid core for the nascent apoB to encircle and wrap around it [11]. It is thought that the fusion of immature VLDL with lipidic vesicles derived from smooth ER regions (separation of rough and smooth ER membrane regions is indicated in the figure by ][) induces the maturation and secretion of VLDL from the hepatocytes into the blood. **B. HCV assembly.** HCV core protein localizes to the surface of lipid droplets and is able to interact with viral structural proteins assembled on the ER. Furthermore, intracellular membranes containing the HCV replication complex are enriched in MTP, apoB, and also apoE, and inhibition of the expression or activity of either of these factors blocks the release of infectious HCV. Thus, the release of infectious HCV may depend on virions being packaged as a VLDL-like particle.

HCV is a small, enveloped virus with a single-stranded RNA genome of positive polarity that replicates primarily, if not exclusively, within hepatocytes [2]. Its 9.6-kb RNA genome is translated by cellular ribosomes to yield a single polyprotein of approximately 3,000 amino acids, which is cleaved by host and viral proteases into the final gene products: core, the glycoproteins E1 and E2, p7, and the non-structural (NS) proteins NS2, NS3, NS4A, NS4B, NS5A, NS5B, and possibly an alternative reading frame protein termed ARFP [2]. Core, E1, and E2 are the structural components of the viral particle, whereas the NS gene products NS3 to NS5B mediate genome replication, a process that occurs exclusively in the cytoplasm [2]. Progeny virions are thought to bud at intracellular membranes and egress via the cellular secretory pathway before going on to infect naïve cells [3]. HCV infection alters cellular metabolism, and in particular lipid homeostasis, with important pathological consequences [4]. HCV infection has experimentally been shown to augment lipogenesis, and is clinically linked to steatosis. These HCV-induced metabolic changes may be important for HCV replication and in particular the HCV assembly process, which is closely linked to the hepatic triglyceride and VLDL metabolism and may even depend on it [5] (Figure 1). Indirect evidence for a link between cellular triglyceride metabolism and HCV assembly is the fact that in patient sera, the virus exists in different morphological forms. These forms include more classical virions, which are thought to consist of viral capsids containing the viral genome surrounded by a lipid bilayer into which the viral envelope glycoprotein complexes are supposed to be incorporated, as well as forms in which the virus associates with lipoproteins [6–8]. While only very few information on structural and biochemical characterization of lipoprotein-associated virus is available, lipoprotein association has profound impact on the viral life cycle of HCV as it is thought to increase the infectious properties of the virion and to shield it from immune responses [9,10]. Whether and how HCV-glycoprotein mediated cell entry is altered by the association of the virus with lipoproteins is an issue that is being actively investigated and that may have important consequences on development of future treatment and therapeutics.

HCV has been reported to transmit frequently by direct exposure to blood products, but the virus has also been shown to transmit horizontally [12]. Upon exposure to the virus, it remains unclear how the virus transfers from the bloodstream through the liver endothelium into the liver in order to gain access to its primary site of replication, the hepatocytes. In contrast, analysis of HCV ‘hepatocyte’ entry has made impressive progress over the last few years with the development and availability of *in vitro* HCV infection systems and the identification of the minimal set of required HCV co-receptors.

This review gives a very brief outline of the different forms of infectious virions detected in plasma and describes in detail the mechanisms and roles of cellular co-factors in HCV cell entry.

## 2. HCV Exists in Lipoprotein-Associated Forms

Many lines of evidence point to an important role of the very low density lipoprotein (VLDL) biosynthesis machinery in the HCV assembly process. Indeed, in patient serum HCV is detected in varying forms [7]. Lipoprotein-free or -poor forms of HCV are thought to consist of particles of 50 to 60 nm diameter surrounded by a lipid bilayer containing the HCV glycoprotein complexes [13]; but HCV has also been shown to exist in association with lipoproteins, and this association is thought to increase HCV specific infectivity significantly [6,7,9,14,15]. The molecular determinants that lead to secretion of HCV that is associated with lipoproteins are not clear, and data on the links between the HCV- and the VLDL-assembly machinery remain contradictory. On one hand, it has been reported that HCV core protein localizes to the surface of lipid droplets, that virion assembly depends on the enzymatic activity of microsomal triglyceride transfer protein (MTP) which lipidates nascent apoB lipoprotein [11] and on presence of apoB and apoE [5,16–18] (Figure 1A and B); on the other hand it has been shown that HCV-infection inhibits VLDL assembly [19]. Indeed, chronic HCV infection has been shown to correlate with hypobetalipoproteinemia, e.g., the retention of VLDL in the liver and with steatosis [20]. While the exact links between VLDL and HCV assembly are only starting to be explored, it is clear that an association with lipids evokes the partitioning of HCV on density gradients into two spectra: particles detected at buoyant densities of ca 1.15 g/mL as well as light, lipoprotein containing fractions detected between 1.06 to 1.10 g/mL [6,7,21]. Virions in the light fraction are thought to consist of triglyceride-rich lipoproteins containing apoB, apoE and apoC1, viral nucleocapsids as well as envelope glycoproteins [6,16,17,21–23]. The fact that HCV particles have been shown to contain not only the liver specific apoB100, but also its spliced form apoB48, points to the possibility, that HCV may also originate from the intestine [24]. Overall, the morphology of VLDL associated HCV particles remains elusive. Importantly, the specific infectivity of HCV produced *in vitro* increases significantly upon passage through a chimpanzee or uPA-SCID mice with human liver grafts; and this increase of specific infectivity correlates with an increased portion of lipoprotein-associated virus [9]. Furthermore, infectivity of lipoprotein-associated virus can be neutralized with antibodies targeting several different apolipoproteins, while some antibodies targeted against the viral glycoproteins have been shown to demonstrate reduced neutralization efficiency [10,22,25]. Thus, lipoprotein-association may favor persistent HCV replication by increasing viral specific infectivity and by shielding the virus from humoral immune responses.

## 3. HCV Liver Uptake

**Figure 2:**
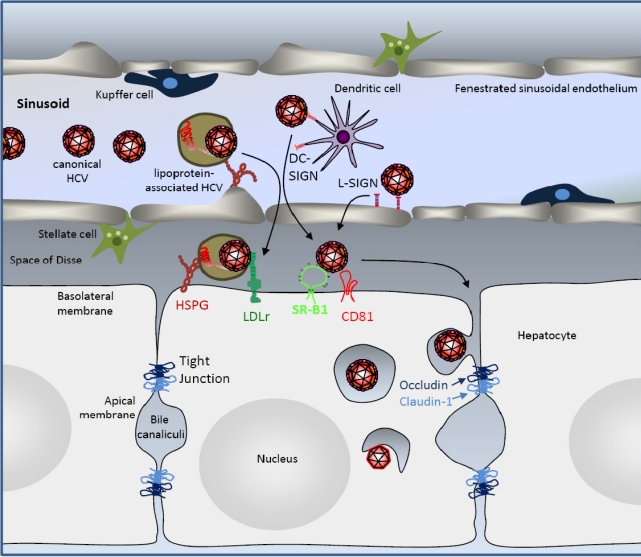
**HCV liver and cell entry.** HCV circulates in the blood in canonical and lipoprotein-associated forms. To cross from the fenestrated sinusoidal liver endothelium into the space of Disse, HCV may diffuse through fenestrae or it may be captured and transcytosed by C-type lectins L-SIGN or DC-SIGN, expressed on the liver endothelium and dentritic cells, respectively. Alternatively, heparan sulfate proteoglycans (HSPG) on the surface of endothelial cells may favor the transfer of canonical and lipoprotein-associated virus into the liver. In the liver, canonical HCV hepatocytes entry requires the cellular entry factors scavenger receptor BI (SR-BI), the tetraspanin CD81 as well as Occludin and Claudin-1, tight junction factors that play a role in the physical separation of basolateral and apical membranes. HSPG and low density lipoprotein receptor (LDLr) have also been implied in HCV cell entry, but whether their roles apply particularly to lipoprotein-associated HCV remains unclear. HCV-virions are internalized into hepatocytes by clathrin-mediated endocytosis and overall the roles of the various HCV entry factors in cell attachment, internalization and fusion are not yet elucidated. Please refer to the text for further details.

In order to establish productive and persistent infection within its primary target, the hepatocytes, HCV is thought to need to transfer from the bloodstream through the liver endothelium into the liver at very early stages of infection. How uptake of HCV into the liver occurs, and whether lipoprotein association can modulate this process remains unclear. The capillary liver endothelium plays a central and active role in regulating the exchange of macromolecules, solutes and fluid between the blood and liver tissue. Transport across the liver endothelium appears to be a very complex process in which the substances are transported according to their size, charge and chemistry. Besides endocytosis and transcytosis, endothelial transport in the liver sinusoidal endothelium occurs through fenestrae. Liver sinusoidal endothelial cells (LSEC)-fenestrae measure between 100 and 200 nm in diameter, and appear to be membrane bound round cytoplasmic holes [26]. Their morphology resembles that of a sieve, suggesting their filtration effect [27]. HCV may traverse the liver endothelium by passive diffusion through the fenestrae, or by active transcytosis through the liver endothelium. Active transcytosis is generally the more favoured hypothesis for three reasons. Firstly, hepatic sinusoidal endothelial cells have a remarkable capacity to internalize and process a diverse range of antigens. Secondly, in the inflammatory state passive diffusion may be limited due to the expression of different classes of adhesion molecules including ICAM-1, VCAM-1, *etc.*, that attract and favour adhesion of leukocytes to the liver endothelium. Finally in inflammatory states the fenestrae of the liver endothelium tend to ‘close up’ [28]. Indeed, a set of capture receptors called C-type lectins that are expressed on liver endothelium and/or dendritic cells have previously been shown to mediate uptake of viruses of several different genera, resulting either in transcytosis of the virus across the liver endothelial barrier [29–31] or resulting in transfer and transmission of virus to its proper host cells. Indeed, DC-SIGN-mediated enhancement of HIV infection is not limited to concentrating viral particles onto the cell surface, but involves internalization and complex intracellular trafficking of virions [32,33]. DC-SIGN and L-SIGN recognize high-mannose carbohydrates on the surface of ligands [34–36] and a large body of evidence suggests that carbohydrate recognition is the sole determinant of their ligand specificity [34–37]. However, while L-SIGN is strictly high-mannose carbohydrate specific, DC-SIGN can interact with other carbohydrates. These differences in carbohydrate specificity account for differences in the ligand spectrum of these lectins [37,38]. It has been shown that both L-SIGN and DC-SIGN can interact with the HCV glycoproteins and capture and transmit HCV to adjacent hepatocytes [39–42]. L-SIGN and DC-SIGN are expressed on LSECs and dendritic cells, respectively, *in vivo*, and important questions that remain to be answered are whether, by which mechansims and how efficiently these lectins may transfer HCV across the endothelium (Figure 2).

Besides a direct recognition and transfer by C-type lectins, HCV particles may cross the epithelium in conjunction with lipoproteins. After secretion from the liver, triglycerides in the cores of triglyceride-rich lipoproteins (TRLs) such as VLDL are hydrolyzed by lipoprotein lipase (Lpl), resulting in the formation of intermediate-density lipoproteins (IDLs). Lpl can remain associated with lipoprotein particles, but most of the enzyme associates with heparan sulfate proteoglycans (HSPG) on the surface of endothelial cells [43,44]. The mechanism of hepatic clearance of TRLs remains controversial. The particles may diffuse through fenestrae or are transcytosed through the endothelium and then sequester in the liver perisinusoidal space (space of Disse), where they undergo further processing by hepatic lipase (HL) and Lpl, both of which are detectable in the space of Disse and may remain associated with TRLs [45,46]. Lipoprotein lipase and hepatic lipase may furthermore have additional functions by serving as bridging factors for receptor-mediated lipoprotein uptake. However, lipoprotein lipase mediated uptake of HCV has recently been shown to result in non-productive infection [47]. On the hepatocytes surface, multiple receptors mediating clearance of TRL remnants and their lysosomal catabolism appear to exist. These include the low density lipoprotein receptor (LDLr), the LDLR-related protein (LRP), scavenger receptors (SR) and cell-surface HSPGs [48].

Besides sinusoidal endothelial cells, two additional non parenchymal liver-resident cell types, line the walls of hepatic sinusoids: Kupffer cells and hepatic stellate cells (Figure 2). In addition, intrahepatic lymphocytes are often present in the sinusoidal lumen. The role of these different cell types in liver and cell entry of canonical or lipoprotein-associated HCV has so far not been investigated. Interestingly, one report suggests that B cells may serve as a means for HCV to cross the liver endothelial barrier. Indeed, cell culture produced HCV has been shown to associate and to be endocytosed by B cells and B cell–associated virus readily transmits to and infects hepatoma cells, showing an enhanced infectivity compared with extracellular virus [49].

## 4. Hepatocyte Uptake of HCV Particles

The infection process begins with the attachment of a virus to the surface of its host cell and is usually mediated by an envelope glycoprotein complex that is anchored into the surface of the viral particle. With the availability of the first HCV *in vitro* infection systems [13,50–54], it has become clear at least in the context of HCV particles of canonical density, which are lipoprotein-free or -poor, that the HCV glycoproteins are required for the cell entry process and mediate cell attachment, virion internalization and fusion [13,50]. Whether this holds true for lipoprotein-associated or -enriched forms of HCV, is currently being investigated. Two HCV *in vitro* infection systems are predominantly used to study HCV cell entry. The first system, HCV pseudoparticles (HCVpp), consists of retroviral core particles surrounded by a lipid bilayer containing functional HCV E1E2 complexes [50–52]. Into the retroviral core, a retroviral genome encoding marker genes for fluorescent, luminescent or selective detection can be incorporated [55]. The most prominent features of HCVpp are that they can be engineered to display the HCV glycoproteins of any viral genotype and that they mediate an abortive infection cycle and allow the integration of various marker genes. The second system consists of replicative HCV particles termed HCVcc, which are produced from a particular HCV genome, isolated from a fulminant Japanese hepatitis case. This viral isolate is the only one known to replicate productively *in vitro* [13,53,54]. With the development of this system, study of the complete HCV life cycle has finally become possible.

Like for many other viruses, glycosaminoglycans seem to be an initial docking site for HCV attachment [56,57]. After the initial attachment to its host cell, a virus generally binds to specific cellular entry factors, which are responsible for initiating a series of events that eventually lead to the release of the viral genome into the cytosol. A number of cellular factors have been shown to be required for the HCV cell entry process, but their exact roles remain unclear. These comprise the tetraspanin CD81, the lipoprotein receptor scavenger receptor BI (SR-BI) as well as two tight junction factors called claudin 1 (CLDN) and occluding (OCLN).

### 4.1. The Tetraspanin CD81

The first HCV entry factor shown to be required for HCV cell entry was the tetraspanin CD81 [58]. CD81 is a member of the tetraspanin superfamily and very ubiquitiously expressed. Key feature of tetraspanins is the formation of an extended network at the cell surface, which is thought to structure the membrane by coordinating homologous, autologous and heterologous protein interactions. Such ‘tetraspanin-enriched microdomains’ (TEMs) are emerging to play important roles in cellular signalling, cytoskeletal reorganization, migration, adhesion, fusion and proliferation; but very recently the association of CD81 with TEMs has been shown not to be required for HCV cell entry [59]. Tetraspanins have overall no intrinsic enzymatic activity and are rather thought to function as adaptor proteins by sorting and modulating localization and interactions of membrane resident proteins. Infection of primary human hepatocytes or hepatoma cell lines was inhibited by anti CD81 antibodies [13,51,53,60,61] and after downregulation of CD81 using an siRNA approach [62]. HepG2 and HH29 cells, hepatoma cell lines, which do not express endogenously CD81, become permissive to HCVpp of all genotypes upon ectopic CD81 expression [60,62,63]. These data were confirmed with HCVcc [13,53]. At which step of the HCV cell entry process CD81 is involved remains unclear. Solubilized E2 protein can bind to CD81 and a recombinant expressed form of the large extracellular loop of CD81 can bind to HCV [58], suggesting that CD81 may be a binding factor. However, several laboratories have shown that in receptor competition assays, anti-CD81 antibodies can inhibit infection to similar extents whether added before, with or after virus-cell incubation, suggesting that either the primary role of CD81 is not to mediate binding of the virus to the cell surface [64,65], or that the role of CD81 extends beyond. Interestingly, a protein found to interact with CD81, which is expressed ubiquitiously but not in hepatocytes, has been found to inhibit HCV-CD81 interactions and to block HCV cell entry in a dominant-negative fashion [66]. Furthermore, HCV has been shown to spread by cell-to-cell transmission, a process that can occur independently of CD81 [67,68]. The importance of direct cell-to-cell transmission of HCV *in vivo*, within the infected liver, remains to be evaluated.

### 4.2. The Scavenger Receptor BI

The scavenger receptor class B type I (SR-BI), also called CLA-1, functions as lipoprotein receptor that mediates selective uptake of cholesteryl ester from apoA containing lipoproteins and in particular high density lipoprotein (HDL), via a two-step mechanism involving the binding of lipoproteins to its extracellular domain followed by lipid uptake [69]. SR-BI was identified as co-receptor for HCV cell entry in binding assays with a soluble form of E2 glycoprotein (sE2) [70]. SR-BI recognition by sE2 required the hyper variable region 1 of E2 (HVR1) and antibodies against SR-BI were shown to inhibit HCV infection [60], suggesting an important role for SR-BI in HCV cell entry. Interestingly, kinetics of inhibition with anti-SR-BI and anti-CD81 antibodies suggest that SR-BI might act concomitantly with CD81 [64], but the fact that HCVcc bind SR-BI expressing CHO cells but not CD81 expressing CHO cells [71] may imply that a first contact with SR-BI is necessary before the viral particle can interact with CD81. An important role for SR-BI in HCV entry was confirmed by observations that specific ligands to SR-BI such as high density lipoprotein (HDL) and oxidized low density lipoprotein modulate the infectivity of HCV [72–74]. With the establishment of functional complementation assays it has now become clear that SR-BI and its physiological lipid transfer activity are required for HCV cell entry [75,76]. Mutational analysis of SR-BI in receptor complementation assays has shown that the intracellular C-terminal cytoplasmic tail of SR-BI modulates HCV infection. A C-terminal deletion mutant, expression of the spliced form SR-BII which displays an alternative C-terminal tail or the replacement of the SR-BI cytoplasmic tail in an SR-BI/CD36 chimera all reduced HCV infection. These data suggest that the C-terminal tail of SR-BI does influence the HCV cell entry process. Whether the C-terminal tail modulates intracellular trafficking, membrane localization or other mechanisms remains to be seen. Interestingly, interference with the interaction of SR-BI with PDZK1, an enzyme that regulates localization, assembly and scaffolding of SR-BI [77], by mutation of the AKL motif in the C-terminus of SR-BI, did not affect HCV entry. Mutational analysis of SR-BI in receptor competition assays showed that the lipid transfer properties of SR-BI are required for HCV cell entry. SR-BI takes up cholesterol ester (CE) from low and high density lipoproteins via a two-step mechanism. In the case of HDL, it has been shown that SR-BI binds HDL with high-affinity and mediates lipid uptake by transferring the lipids to the cholesterol pool of the target cell membrane [69,78].

### 4.3. The Tight Junction Factors Claudin-1 and Occludin

Besides SR-BI and CD81, two additional factors that are essential for HCV cell entry - probably at late stages - have been identified by expression cloning. These are claudin-1 (CLDN1) and occludin (OCLN) [71,76]. In addition, Claudin-6 and -9 have been shown to enable HCV entry into nonpermissive cells in replacement of Claudin-1 [79]. CLDN1 and OCLN are two factors that are expressed at the interface between basolateral and apical membranes. At this location, they form part of tight-junctions that regulate paracellular transport of solutes, water and ions and separate the space of Disse from the bile canaliculi thus generating apical-basal cell polarity (see Figure 2). With the discovery of TJ proteins as entry factors, the role of cell differentiation and polarization in the HCV cell entry process have become important issues. On the one hand the dependence of HCV assembly on an active VLDL assembly machinery suggests indirectly that cell differentiation is important for HCV production, because VLDL assembly is a metabolic function that characterizes differentiated hepatocytes. On the other hand, HCV infection has been shown to provoke the downregulation of CLDN1 and OCLN expression and TJ function and to induce depolarization of infected hepatocytes with potential pathological consequences [80,81]. The negative effect of HCV infection on OCLN expression has been shown to be due to the induction of vascular endothelial growth factor [82].

In addition, the roles of CLDN1 and OCLN in HCV cell entry imply that parallels between HCV and coxsackievirus B (CVB) cell entry may exist. CVB interacts with its primary receptor decay accelerating factor, and this interaction induces lateral movement of the virus-receptor complex towards TJs, where the binding of the virus to coxsackie-adenovirus receptor triggers virus internalization [83]. In a similar fashion, an interesting set of data suggests that upon engagement with HCV, CD81 triggers intracellular signaling responses that lead to actin remodeling and relocalization of CD81 away from the basolateral membrane towards TJs [84]. Thus CD81 may function as a shuttle to transfer the virus from the basolateral surface towards TJs. The actual roles of CLDN1 and OCLN in HCV cell entry remain unclear, but interestingly a direct interaction between the HCV envelope glycoproteins and OCLN has been shown [85]. Furthermore, knock-down of OCLN in a cell-cell fusion assay, where fusion activity depends on cell surface expression of the HCV glycoprotein complex, diminished fusion activity, suggesting that OCLN may be implied in the HCV fusion process [86]. Receptor complementation with deleted or mutated versions of CLDN1 demonstrated that the extracellular loop 1 of claudin-1 [71] and more particularly a highly conserved motif required for cell-cell contact formation is required for HCV entry [87]. Finally, downregulation of other TJ factors such as junctional adhesion molecule A or zonula occludens protein 1 did not affect HCV cell entry [86].

### 4.4. The Route of HCV Cell Entry

The uptake of HCV into hepatocytes has been shown to be dependent on clathrin-mediated endocytosis [88], and experimental evidence based on drugs that inhibit acidification of endosomes have furthermore shown that low pH in endosomes is required for the HCV cell entry process [60,89]. With the set up of *in vitro* fusion assays, based on liposomes that do not display any cellular factors, it was furthermore shown that HCV fuses with liposomes in a low pH-dependent manner [90]. Curiously, low pH exposure of HCV virions does not inactivate the function of the glycoprotein complex in cell entry and fusion, suggesting that besides low pH exposure additional triggers or modifications are required to activate the fusion machinery of the HCV glycoprotein complexes [89]. HCV internalization before and after the uncoating process has furthermore been shown to depend on intact microtubules and their dynamic turnover [91].

## 5. Contribution of VLDL to Hepatocyte Uptake of HCV

Besides CD81, SR-BI, CLDN and OCLN, a potential involvement of the low density lipoprotein receptor (LDLr) in HCV cell entry has been reported by many groups. The potential involvement of LDLr in HCV entry originated with the observation that HCV infection is associated with mixed cryoglobulinemia, where VLDL is selectively associated with HCV in type II cryoglobulin complexes [92]. This observation, together with the finding that anti-apoB is associated with HCV in infected serum, suggested that LDLr may be a receptor for HCV complexed to VLDL or LDL [8,10,16,23,93]. While in the cell entry process of lipoprotein-free or -poor HCV, so far not much evidence has pointed to a role for the LDLr, this scenario has started to change dramatically with the availability of *in vitro* produced authentic HCV particles that can be recovered in light, lipoprotein containing fractions of density gradients. Indeed, it has been shown that endocytosis of HCV correlates with the presence of LDLr and its activity by performing receptor competition assays with anti LDLr antibodies, with anti-apoB and apoE antibodies as well as with VLDL and LDL [10,16,18,23,27,93]. Furthermore, knock down of LDLr in primary hepatocytes has been shown to interfere with HCV infection [61]. The physiological role of the LDLr is to transport cholesterol-containing lipoprotein particles from the extracellular medium into cells. The most important physiological ligand for the LDLr is the apoB100 containing low density lipoprotein (LDL). In addition, the receptor also exhibits high-affinity binding of lipoproteins that contain multiple copies of apoE, like some forms of VLDL and certain intermediate density lipoproteins. Receptor ligand complexes enter cells by endocytosis via clathrin-coated pits and are delivered to endosomes, where low pH triggers release of bound lipoprotein particles. Released lipoprotein particles proceed to lysosomes, where the cholesterol esters are hydrolyzed to free cholesterol [94].

Thus interactions between apoB or apoE associated with HCV and the LDLr may be involved in liver uptake as well as initial attachment to the hepatocyte cell surface. These processes may be further modified by Lpl, which has been shown to be involved in HCV cell entry, albeit in a non-productive fashion [47]. Non-specific cell attachment may favor subsequent interaction of the HCV glycoproteins with CD81, trafficking of this complex towards TJs, followed by internalization and fusion. To what extent lipoprotein-association and lipidation status of the lipoprotein/virus complex alters or modifies the requirement for the HCV co-receptors or modulates their respective roles, remains to be seen.

## 6. Conclusions

The association of HCV with lipoproteins is thought to serve as a mechanism to increase infection and to escape immune detection and help the virus to maintain persistent infection over time. It may also help, at least in part, to explain the hepatotropism of HCV. Indeed, apolipoproteins regulate the uptake of lipid particles to the liver. At the same time does the association of HCV with lipoproteins pose technical challenges and limitations to the research field. Biochemical isolation and characterization of lipoprotein associated virus is proving difficult and production efficiency of HCV and HCV lipoprotein-association vary furthermore with cell culture conditions. Thus comparative analysis of *in vitro* produced forms of HCV is an important but difficult task. The use of HCV derived from patient sera in *in vitro* infection systems is notoriously difficult due to the genetic and morphological heterogeneity of HCV, which may in part be determined by the metabolic predisposition of its host. But with novel cell culture systems that support survival of differentiated, polarized hepatocytes cultures, which are metabolically active for prolonged periods of time, studying the importance of the liver lipid metabolism in the HCV life cycle and particularly the cell entry process may become more feasible.

## References

[1] Alter MJ (2007). Epidemiology of hepatitis C virus infection. World J Gastroenterol.

[2] Moradpour D, Penin F, Rice CM (2007). Replication of hepatitis C virus. Nat Rev Microbiol.

[3] Lindenbach BD, Thiel HJ, Rice CM, Knipe DM, Howley PM (2007). Flaviviridae: The Viruses and Their Replication. Fields Virology.

[4] Negro F (2006). Mechanisms and significance of liver steatosis in hepatitis C virus infection. World J Gastroenterol.

[5] Miyanari Y, Atsuzawa K, Usuda N, Watashi K, Hishiki T, Zayas M, Bartenschlager R, Wakita T, Hijikata M, Shimotohno K (2007). The lipid droplet is an important organelle for hepatitis C virus production. Nat Cell Biol.

[6] Thomssen R, Bonk S, Propfe C, Heermann KH, Kochel HG, Uy A (1992). Association of hepatitis C virus in human sera with beta-lipoprotein. Med Microbiol Immunol.

[7] Bradley D, McCaustland K, Krawczynski K, Spelbring J, Humphrey C, Cook EH (1991). Hepatitis C virus: Buoyant density of the factor VIII-derived isolate in sucrose. J Med Virol.

[8] Andre P, Komurian-Pradel F, Deforges S, Perret M, Berland JL, Sodoyer M, Pol S, Brechot C, Paranhos-Baccala G, Lotteau V (2002). Characterization of low- and very-low-density hepatitis C virus RNA-containing particles. J Virol.

[9] Lindenbach BD, Meuleman P, Ploss A, Vanwolleghem T, ASyder AJ, McKeating JA, Lanford RE, Feinstone SM, Major ME, Leroux-Roels G, Rice CM (2006). Cell culture-grown hepatitis C virus is infectious *in vivo* and can be recultured *in vitro*. Proc Natl Acad Sci USA.

[10] Maillard P, Huby T, Andreo U, Moreau M, Chapman J, Budkowska A (2006). The interaction of natural hepatitis C virus with human scavenger receptor SR-BI/Cla1 is mediated by ApoB-containing lipoproteins. FASEB J.

[11] Hussain MM, Shi J, Dreizen P (2003). Microsomal triglyceride transfer protein and its role in apoB-lipoprotein assembly. J Lipid Res.

[12] Diaz O, Cubero M, Trabaud MA, Quer J, Icard V, Esteban JI, Lotteau V, Andre P (2008). Transmission of low-density hepatitis C viral particles during sexually transmitted acute resolving infection. J Med Virol.

[13] Wakita T, Pietschmann T, Kato T, Date T, Miyamoto M, Zhao Z, Murthy K, Habermann A, Krausslich HG, Mizokami M, Bartenschlager R, Liang TJ (2005). Production of infectious hepatitis C virus in tissue culture from a cloned viral genome. Nat Med.

[14] Nielsen SU, Bassendine MF, Burt AD, Martin C, Pumeechockchai W, Toms GL (2006). Association between hepatitis C virus and very-low-density lipoprotein (VLDL)/LDL analyzed in iodixanol density gradients. J Virol.

[15] Nielsen SU, Bassendine MF, Martin C, Lowther D, Purcell PJ, King BJ, Neely D, Toms GL (2008). Characterization of hepatitis C RNA-containing particles from human liver by density and size. J Gen Virol.

[16] Gastaminza P, Cheng G, Wieland S, Zhong J, Liao W, Chisari FV (2008). Cellular determinants of hepatitis C virus assembly, maturation, degradation, and secretion. J Virol.

[17] Chang KS, Jiang J, Cai Z, Luo G (2007). Human apolipoprotein e is required for infectivity and production of hepatitis C virus in cell culture. J Virol.

[18] Owen DM, Huang H, Ye J, Gale M (2009). Apolipoprotein E on hepatitis C virion facilitates infection through interaction with low-density lipoprotein receptor. Virology.

[19] Perlemuter G, Sabile A, Letteron P, Vona G, Topilco A, Chretien Y, Koike K, Pessayre D, Chapman J, Barba G, Brechot C (2002). Hepatitis C virus core protein inhibits microsomal triglyceride transfer protein activity and very low density lipoprotein secretion: A model of viral-related steatosis. FASEB J.

[20] Alaei M, Negro F (2008). Hepatitis C virus and glucose and lipid metabolism. Diabetes Metab.

[21] Thomssen R, Bonk S, Thiele A (1993). Density heterogeneities of hepatitis C virus in human sera due to the binding of beta-lipoproteins and immunoglobulins. Med Microbiol Immunol.

[22] Meunier JC, Russell RS, Engle RE, Faulk KN, Purcell RH, Emerson SU (2008). Apolipoprotein c1 association with hepatitis C virus. J Virol.

[23] Huang H, Sun F, Owen DM, Li W, Chen Y, Gale M, Ye J (2007). Hepatitis C virus production by human hepatocytes dependent on assembly and secretion of very low-density lipoproteins. Proc Natl Acad Sci USA.

[24] Diaz O, Delers F, Maynard M, Demignot S, Zoulim F, Chambaz J, Trepo C, Lotteau V, Andre P (2006). Preferential association of Hepatitis C virus with apolipoprotein B48-containing lipoproteins. J Gen Virol.

[25] Martin C, Nielsen SU, Ibrahim S, Bassendine MF, Toms GL (2008). Binding of liver derived, low density hepatitis C virus to human hepatoma cells. J Med Virol.

[26] Daneker GW, Lund SA, Caughman SW, Swerlick RA, Fischer AH, Staley CA, Ades EW (1998). Culture and characterization of sinusoidal endothelial cells isolated from human liver. In Vitro Cell Dev Biol Anim.

[27] Braet F, Riches J, Geerts W, Jahn KA, Wisse E, Frederik P (2009). Three-dimensional organization of fenestrae labyrinths in liver sinusoidal endothelial cells. Liver Int.

[28] Yokomori H (2008). New insights into the dynamics of sinusoidal endothelial fenestrae in liver sinusoidal endothelial cells. Med Mol Morphol.

[29] Breiner KM, Schaller H, Knolle PA (2001). Endothelial cell-mediated uptake of a hepatitis B virus: A new concept of liver targeting of hepatotropic microorganisms. Hepatology.

[30] Bobardt MD, Chatterji U, Selvarajah S, Van der Schueren B, David G, Kahn B, Gallay PA (2007). Cell-free human immunodeficiency virus type 1 transcytosis through primary genital epithelial cells. J Virol.

[31] Zhu ZB, Makhija SK, Lu B, Wang M, Rivera AA, Preuss M, Zhou F, Siegal GP, Alvarez RD, Curiel DT (2004). Transport across a polarized monolayer of Caco-2 cells by transferrin receptor-mediated adenovirus transcytosis. Virology.

[32] Kwon DS, Gregorio G, Bitton N, Hendrickson WA, Littman DR (2002). DC-SIGN-mediated internalization of HIV is required for trans-enhancement of T cell infection. Immunity.

[33] McDonald MC, Dhadly P, Cockerill GW, Cuzzocrea S, Mota-Filipe H, Hinds CJ, Miller NE, Thiemermann C (2003). Reconstituted high-density lipoprotein attenuates organ injury and adhesion molecule expression in a rodent model of endotoxic shock. Shock.

[34] Lin G, Simmons G, Pohlmann S, Baribaud F, Ni H, Leslie GJ, Haggarty BS, Bates P, Weissman D, Hoxie JA, Doms RW (2003). Differential N-linked glycosylation of human immunodeficiency virus and Ebola virus envelope glycoproteins modulates interactions with DC-SIGN and DC-SIGNR. J Virol.

[35] Feinberg H, Mitchell DA, Drickamer K, Weis WI (2001). Structural basis for selective recognition of oligosaccharides by DC-SIGN and DC-SIGNR. Science.

[36] Appelmelk BJ, van Die I, van Vliet SJ, Vandenbroucke-Grauls CM, Geijtenbeek TB, van Kooyk Y (2003). Cutting edge: Carbohydrate profiling identifies new pathogens that interact with dendritic cell-specific ICAM-3-grabbing nonintegrin on dendritic cells. J Immunol.

[37] Guo Y, Feinberg H, Conroy E, Mitchell DA, Alvarez R, Blixt O, Taylor ME, Weis WI, Drickamer K (2004). Structural basis for distinct ligand-binding and targeting properties of the receptors DC-SIGN and DC-SIGNR. Nat Struct Mol Biol.

[38] Van Liempt E, Imberty A, Bank CM, Van Vliet SJ, Van Kooyk Y, Geijtenbeek TB, Van Die I (2004). Molecular basis of the differences in binding properties of the highly related C-type lectins DC-SIGN and L-SIGN to Lewis X trisaccharide and Schistosoma mansoni egg antigens. J Biol Chem.

[39] Cormier EG, Durso RJ, Tsamis F, Boussemart L, Manix C, Olson WC, Gardner JP, Dragic T (2004). L-SIGN (CD209L) and DC-SIGN (CD209) mediate transinfection of liver cells by hepatitis C virus. Proc Natl Acad Sci USA.

[40] Gardner JP, Durso RJ, Arrigale RR, Donovan GP, Maddon PJ, Dragic T, Olson WC (2003). L-SIGN (CD 209L) is a liver-specific capture receptor for hepatitis C virus. Proc Natl Acad Sci USA.

[41] Lozach PY, Amara A, Bartosch B, Virelizier JL, Arenzana-Seisdedos F, Cosset FL, Altmeyer R (2004). C-type lectins L-SIGN and DC-SIGN capture and transmit infectious hepatitis C virus pseudotype particles. J Biol Chem.

[42] Falkowska E, Durso RJ, Gardner JP, Cormier EG, Arrigale RA, Ogawa RN, Donovan GP, Maddon PJ, Olson WC, Dragic T (2006). L-SIGN (CD209L) isoforms differently mediate trans-infection of hepatoma cells by hepatitis C virus pseudoparticles. J Ge Virol.

[43] Williams KJ, Fless GM, Petrie KA, Snyder ML, Brocia RW, Swenson TL (1992). Mechanisms by which lipoprotein lipase alters cellular metabolism of lipoprotein(a), low density lipoprotein, and nascent lipoproteins. Roles for low density lipoprotein receptors and heparan sulfate proteoglycans. J Biol Chem.

[44] Saxena U, Klein MG, Goldberg IG (1991). Identification and characterization of the endothelial cell surface lipoprotein lipase receptor. J Biol Chem.

[45] Mahley RW, Ji ZS (1999). Remnant lipoprotein metabolism: Key pathways involving cell-surface heparan sulfate proteoglycans and apolipoprotein E. J Lipid Res.

[46] Sanan DA, Fan J, Bensadoun A, Taylor JM (1997). Hepatic lipase is abundant on both hepatocyte and endothelial cell surfaces in the liver. J Lipid Res.

[47] Andreo U, Maillard P, Kalinina O, Walic M, Meurs E, Martinot M, Marcellin P, Budkowska A (2007). Lipoprotein lipase mediates hepatitis C virus (HCV) cell entry and inhibits HCV infection. Cell Microbiol.

[48] Cooper AD (1997). Hepatic uptake of chylomicron remnants. J Lipid Res.

[49] Stamataki Z, Shannon-Lowe C, Shaw J, Mutimer D, Rickinson AB, Gordon J, Adams DH, Balfe P, McKeating JA (2009). Hepatitis C virus association with peripheral blood B lymphocytes potentiates viral infection of liver-derived hepatoma cells. Blood.

[50] Bartosch B, Dubuisson J, Cosset FL (2003). Infectious hepatitis C virus pseudo-particles containing functional E1-E2 envelope protein complexes. J Exp Med.

[51] Hsu M, Zhang J, Flint M, Logvinoff C, Cheng-Mayer C, Rice CM, McKeating JA (2003). Hepatitis C virus glycoproteins mediate pH-dependent cell entry of pseudotyped retroviral particles. Proc Natl Acad Sci USA.

[52] Drummer HE, Maerz A, Poumbourios P (2003). Cell surface expression of functional hepatitis C virus E1 and E2 glycoproteins. FEBS Lett.

[53] Lindenbach BD, Evans MJ, Syder AJ, Wolk B, Tellinghuisen TL, Liu CC, Maruyama T, Hynes RO, Burton DR, McKeating JA, Rice CM (2005). Complete replication of hepatitis C virus in cell culture. Science.

[54] Zhong J, Gastaminza P, Cheng G, Kapadia S, Kato T, Burton DR, Wieland SF, Uprichard SL, Wakita T, Chisari FV (2005). Robust hepatitis C virus infection *in vitro*. Proc Natl Acad Sci USA.

[55] Bartosch B, Cosset FL (2004). Strategies for retargeted gene delivery using vectors derived from lentiviruses. Curr Gene Ther.

[56] Barth H, Schafer C, Adah MI, Zhang F, Linhardt RJ, Toyoda H, Van Kuppevelt TH, Depla E, Von Weizsacker F, Blum HE, Baumert TF (2003). Cellular binding of hepatitis C virus envelope glycoprotein E2 requires cell surface heparan sulfate. J Biol Chem.

[57] Germi R, Crance JM, Garin D, Guimet J, Lortat-Jacob H, Ruigrok RW, Zarski JP, Drouet E (2002). Cellular glycosaminoglycans and low density lipoprotein receptor are involved in hepatitis C virus adsorption. J Med Virol.

[58] Pileri P, Uematsu Y, Campagnoli S, Galli G, Falugi F, Petracca R, Weiner AJ, Houghton M, Rosa D, Grandi G, Abrignani S (1998). Binding of hepatitis C virus to CD81. Science.

[59] Rocha-Perugini V, Lavie M, Delgrange D, Canton J, Pillez A, Potel J, Lecoeur C, Rubinstein E, Dubuisson J, Wychowski C, Cocquerel L (2009). The association of CD81 with tetraspanin-enriched microdomains is not essential for Hepatitis C virus entry. BMC Microbiol.

[60] Bartosch B, Vitelli A, Granier C, Goujon C, Dubuisson J, Pascale S, Scarselli E, Cortese R, Nicosia A, Cosset FL (2003). Cell entry of hepatitis C virus requires a set of co-receptors that include the CD81 tetraspanin and the SR-B1 scavenger receptor. J Biol Chem.

[61] Molina S, Castet V, Fournier-Wirth C, Pichard-Garcia L, Avner R, Harats D, Roitelman J, Barbaras R, Graber P, Ghersa P, Smolarsky M, Funaro A, Malavasi F, Larrey D, Coste J, Fabre JM, Sa-Cunha A, Maurel P (2007). The low-density lipoprotein receptor plays a role in the infection of primary human hepatocytes by hepatitis C virus. J Hepatol.

[62] Zhang J, Randall G, Higginbottom A, Monk P, Rice CM, McKeating JA (2004). CD81 is required for hepatitis C virus glycoprotein-mediated viral infection. J Virol.

[63] Lavillette D, Tarr AW, Voisset C, Donot P, Bartosch B, Bain C, Patel AH, Dubuisson J, Ball JK, Cosset FL (2005). Characterization of host-range and cell entry properties of the major genotypes and subtypes of hepatitis C virus. Hepatology.

[64] Zeisel MB, Koutsoudakis G, Schnober EK, Haberstroh A, Blum HE, Cosset FL, Wakita T, Jaeck D, Doffoel M, Royer C, Soulier E, Schvoerer E, Schuster C, Stoll-Keller F, Bartenschlager R, Pietschmann T, Barth H, Baumert TF (2007). Scavenger receptor class B type I is a key host factor for hepatitis C virus infection required for an entry step closely linked to CD81. Hepatology.

[65] Koutsoudakis G, Kaul A, Steinmann E, Kallis S, Lohmann V, Pietschmann T, Bartenschlager R (2006). Characterization of the early steps of hepatitis C virus infection by using luciferase reporter viruses. J Virol.

[66] Rocha-Perugini V, Montpellier C, Delgrange D, Wychowski C, Helle F, Pillez A, Drobecq H, Le Naour F, Charrin S, Levy S, Rubinstein E, Dubuisson J, Cocquerel L (2008). The CD81 partner EWI-2wint inhibits hepatitis C virus entry. PLoS One.

[67] Timpe JM, Stamataki Z, Jennings A, Hu K, Farquhar MJ, Harris HJ, Schwarz A, Desombere I, Roels GL, Balfe P, McKeating JA (2008). Hepatit,is C virus cell-cell transmission in hepatoma cells in the presence of neutralizing antibodies. Hepatology.

[68] Witteveldt J, Evans MJ, Bitzegeio J, Koutsoudakis G, Owsianka AM, Angus AG, Keck ZY, Foung SK, Pietschmann T, Rice CM, Patel AH (2009). CD81 is dispensable for hepatitis C virus cell-to-cell transmission in hepatoma cells. J Gen Virol.

[69] Connelly MA, Azhar KS, Abumrad S, Williams DL (1999). Comparison of class B scavenger receptors, CD36 and scavenger receptor BI (SR-BI), shows that both receptors mediate high density lipoprotein-cholesteryl ester selective uptake but SR-BI exhibits a unique enhancement of cholesteryl ester uptake. J Biol Chem.

[70] Scarselli E, Ansuini H, Cerino R, Roccasecca RM, Acali S, Filocamo G, Traboni C, Nicosia A, Cortese R, Vitelli A (2002). The human scavenger receptor class B type I is a novel candidate receptor for the hepatitis C virus. EMBO J.

[71] Evans MJ, von Hahn T, Tscherne DM, Syder AJ, Panis M, Wolk B, Hatziioannou T, McKeating JA, Bieniasz PD, Rice CM (2007). Claudin-1 is a hepatitis C virus co-receptor required for a late step in entry. Nature.

[72] Bartosch B, Verney G, Dreux M, Donot P, Morice Y, Penin F, Pawlotsky JM, Lavillette D, Cosset FL (2005). An interplay between hypervariable region 1 of the hepatitis C virus E2 glycoprotein, the scavenger receptor BI, and high-density lipoprotein promotes both enhancement of infection and protection against neutralizing antibodies. J Virol.

[73] Voisset C, Callens N, Blanchard E, Op De Beeck A, Dubuisson J, Vu-Dac N (2005). High density lipoproteins facilitate hepatitis C virus entry through the scavenger receptor class B type I. J Biol Chem.

[74] von Hahn T, Lindenbach BD, Boullier A, Quehenberger O, Paulson M, Rice CM, McKeating JA (2006). Oxidized low-density lipoprotein inhibits hepatitis C virus cell entry in human hepatoma cells. Hepatology.

[75] Dreux M, Dao Thi VL, Fresquet J, Guerin M, Julia Z, Verney G, Durantel D, Zoulim F, Lavillette D, Cosset FL, Bartosch B (2009). Receptor complementation and mutagenesis reveal SR-BI as an essential HCV entry factor and functionally imply its intra- and extra-cellular domains. PLoS Pathog.

[76] Ploss A, Evans MJ, Gaysinskaya VA, Panis M, You H, de Jong YP, Rice CM (2009). Human occludin is a hepatitis C virus entry factor required for infection of mouse cells. Nature.

[77] Kocher O, Krieger M (2009). Role of the adaptor protein PDZK1 in controlling the HDL receptor SR-BI. Curr Opin Lipidol.

[78] Gu X, TB, Xu S, Acton S, Babitt J, Krieger M (1998). The efficient cellular uptake of high density lipoprotein lipids via scavenger receptor class B type I requires not only receptor-mediated surface binding but also receptor-specific lipid transfer mediated by its extracellular domain. J Biol Chem.

[79] Meertens L, Bertaux C, Cukierman L, Cormier E, Lavillette D, Cosset FL, Dragic T (2008). The tight junction proteins claudin-1, -6, and -9 are entry cofactors for hepatitis C virus. J Virol.

[80] Liu S, Yang W, Shen L, Turner JR, Coyne CB, Wang T (2009). Tight junction proteins claudin-1 and occludin control hepatitis C virus entry and are downregulated during infection to prevent superinfection. J Virol.

[81] Mee CJ, Grove J, Harris HJ, Hu K, Balfe P, McKeating JA (2008). Effect of cell polarization on hepatitis C virus entry. J Virol.

[82] Mee CJ, Farquhar MJ, Harris HJ, Hu K, Ramma W, Ahmed A, Maurel P, Bicknell R, Balfe P, McKeating JA (2009). Hepatitis C virus infection reduces hepatocellular polarity in a vascular endothelial growth factor dependent manner. Gastroenterology.

[83] Coyne CB, Shen L, Turner JR, Bergelson JM (2007). Coxsackievirus entry across epithelial tight junctions requires occludin and the small GTPases Rab34 and Rab5. Cell Host Microbe.

[84] Brazzoli M, Bianchi A, Filippini S, Weiner A, Zhu Q, Pizza M, Crotta S (2008). CD81 is a central regulator of cellular events required for hepatitis C virus infection of human hepatocytes. J Virol.

[85] Benedicto I, Molina-Jimenez F, Barreiro O, Maldonado-Rodriguez A, Prieto J, Moreno-Otero R, Aldabe R, Lopez-Cabrera M, Majano PL (2008). Hepatitis C virus envelope components alter localization of hepatocyte tight junction-associated proteins and promote occludin retention in the endoplasmic reticulum. Hepatology.

[86] Benedicto I, Molina-Jimenez F, Bartosch B, Cosset FL, Lavillette D, Prieto J, Moreno-Otero R, Valenzuela-Fernandez A, Aldabe R, Lopez-Cabrera M, Majano PL (2009). The tight junction-associated protein occludin is required for a postbinding step in hepatitis C virus entry and infection. J Virol.

[87] Cukierman L, Meertens L, Bertaux C, Kajumo F, Dragic T (2009). Residues in a highly conserved claudin-1 motif are required for hepatitis C virus entry and mediate the formation of cell-cell contacts. J Virol.

[88] Blanchard E, Belouzard S, Goueslain L, Wakita T, Dubuisson J, Wychowski C, Rouille Y (2006). Hepatitis C virus entry depends on clathrin-mediated endocytosis. J Virol.

[89] Tscherne DM, Jones CT, Evans MJ, Lindenbach BD, McKeating JA, Rice CM (2006). Time- and temperature-dependent activation of hepatitis C virus for low-pH-triggered entry. J Virol.

[90] Lavillette D, Bartosch B, Nourrisson D, Verney G, Cosset FL, Penin F, Pecheur E (2006). Hepatitis C virus glycoproteins mediate low pH-dependent membrane fusion with liposomes. J Biol Chem.

[91] Roohvand F, Maillard P, Lavergne JP, Boulant S, Walic M, Andreo U, Goueslain L, Helle F, Mallet A, McLauchlan J, Budkowska A (2009). Initiation of hepatitis C virus infection requires the dynamic microtubule network: role of the viral nucleocapsid protein. J Biol Chem.

[92] Agnello V, Abel G (1997). Localization of hepatitis C virus in cutaneous vasculitic lesions in patients with type II cryoglobulinemia. Arthritis Rheum.

[93] Icard V, Diaz O, Scholtes C, Perrin-Cocon L, Ramiere C, Bartenschlager R, Penin F, Lotteau V, Andre P (2009). Secretion of hepatitis C virus envelope glycoproteins depends on assembly of apolipoprotein B positive lipoproteins. PLoS One.

[94] Beglova N, Blacklow SC (2005). The LDL receptor: How acid pulls the trigger. Trends Biochem Sci.

